# Systematic review of locking solutions for non‐tunneled hemodialysis catheters

**DOI:** 10.1111/hdi.13047

**Published:** 2022-10-06

**Authors:** Isabelle Boucley, Auguste Dargent, Pascal Andreu, Jean‐Baptiste Roudaut, François Aptel, Marie Labruyère, Marine Jacquier, Amélie Cransac, Jean‐Pierre Quenot

**Affiliations:** ^1^ Department of Intensive Care University Hospital Dijon Bourgogne Dijon France; ^2^ Hospices Civils de Lyon, Hôpital Edouard Herriot Service de Médecine Intensive‐Réanimation Lyon France; ^3^ Department of Pharmacy University Hospital Dijon Bourgogne Dijon France; ^4^ LNC‐UMR1231 University of Burgundy & Franche Comté Dijon France; ^5^ Lipness Team, INSERM Research Centre LNC‐UMR1231 and LabEx LipSTIC University of Burgundy Dijon France; ^6^ INSERM CIC 1432, Clinical Epidemiology University of Burgundy Dijon France

**Keywords:** intensive care unit, locking solutions, non‐tunneled hemodialysis catheters

## Abstract

**Background:**

We conducted a systematic review of studies investigating lock solutions for use in non‐tunneled hemodialysis catheters.

**Methods:**

We searched PubMed and Cochrane databases from inception to June 11, 2021. Study inclusion criteria were: randomized trial or observational study, adults (>18 years), with acute kidney injury (AKI); and temporary non‐tunneled catheters. We recorded bleeding events, catheter dysfunction and complications.

**Results:**

Of 649 studies identified, 6 were included (4 randomized, 1 non‐randomized trial, 1 retrospective cohort study; sample sizes 78–1496 patients). Citrate was compared to heparin in 4 studies, to saline in 1, and ethanol versus saline in 1. Event‐free survival of non‐tunneled catheters did not differ between groups. Catheter‐related infections and adverse events were less frequent with citrate locks, but reached statistical significance in only two studies.

**Conclusion:**

Existing data are too heterogeneous to enable recommending one type of catheter lock over any other for non‐tunneled hemodialysis catheters.

## INTRODUCTION

Acute kidney injury (AKI) is common in patients in the intensive care unit (ICU), with a prevalence of around 40%, and requires renal replacement therapy (RRT) in almost 20%.^1^ Non‐tunneled dialysis catheters are preferentially used in this population, due to the customarily short duration of RRT, since over 90% of patients recover normal renal function within a few weeks.^2^, ^3^ Conversely, tunneled dialysis catheters are used in patients with chronic kidney failure, either while they are waiting for an arteriovenous fistula, or after failure of arteriovenous fistula. Regardless of the type of catheter (tunneled or non‐tunneled), it is necessary to instill a catheter lock solution into the lumen of the catheter between RRT sessions, to avoid catheter dysfunction (stenosis and/or thrombosis) and infectious complications,^4^, ^5^ which may increase morbidity and mortality as well as hospitalization and resource use. While citrate appears to be the most widely recommended solution for locking tunneled dialysis catheters,^6^ the literature about non‐tunneled dialysis catheters is sparse, and there is no general consensus regarding the optimum locking solution for use in this situation. Unfractionated heparin (UFH) is the gold standard lock solution in France, but the use of UFH in the ICU can be associated with life‐threatening side effects, such as bleeding^7^ or heparin‐induced thrombocytopenia.^8^ Similarly, in 2000, the U.S. Food and Drug Administration (FDA) issued a warning regarding high dose trisodium citrate following a death associated with a mistaken bolus injection of 46.7% citrate. The FDA advised that a 4% citrate solution be used as a safer alternative.^9^, ^10^ Meanwhile, in Europe, the use of high concentrated citrate is still allowed.^11^ To date, there are no consensus‐based international guidelines regarding the lock solution that should be used between two RRT sessions in non‐tunneled hemodialysis catheters, either in the ICU setting or in nephrology.

Our aim was therefore to conduct a systematic review of the literature to gain a deeper understanding of the advantages and disadvantages of different catheter lock solutions for non‐tunneled hemodialysis catheters in patients with AKI.

## METHODS

This review is performed and reported in compliance with the PRISMA Guidelines,^12^ and was registered with PROSPERO under the number CRD42021249631.

### Search and information sources

We searched PubMed and Cochrane databases as well as the French scientific literature database LiSSa (Littérature Scientifique en Santé), from inception to June 11, 2021. The following words were used in the search strategy: “ICU”, “dialysis”, “hemodialysis”, “non‐tunneled”, “catheter”, “lock”, “citrate”, “heparin”, “acute kidney injury”, “renal failure”.

### Study eligibility

We included randomized clinical trials and observational studies. The patients enrolled were all adults (>18 years) with AKI. We included studies with patients admitted to the intensive care unit (ICU) and/or nephrology unit. We placed a restriction on catheter types by including only studies involving patients with temporary non‐tunneled catheters.

Experimental and control interventions consisted of different locking solutions, namely citrate, heparin, saline or ethanol. We excluded studies concerning antibiotic or other coating methods in order to focus only on catheter locks, but we also eliminated studies investigating strategies with antibiotic‐containing lock solutions, to compare exclusively locking solutions made of one active ingredient.

Meaningful outcomes included bleeding events, catheter dysfunction and complications such as catheter‐related bacteraemia (CRB). We interpreted the different definitions of events as given in the included studies.

### Study selection

We downloaded all identified reports into a reference management software that automatically helps to collect and organize research (Zotero for Macintosh). All titles and abstracts were evaluated by two authors for qualification for full‐text screening. A consensus was reached for studies with discordant opinions on the suitability for inclusion.

## RESULTS

### Study selection

**FIGURE 1:**
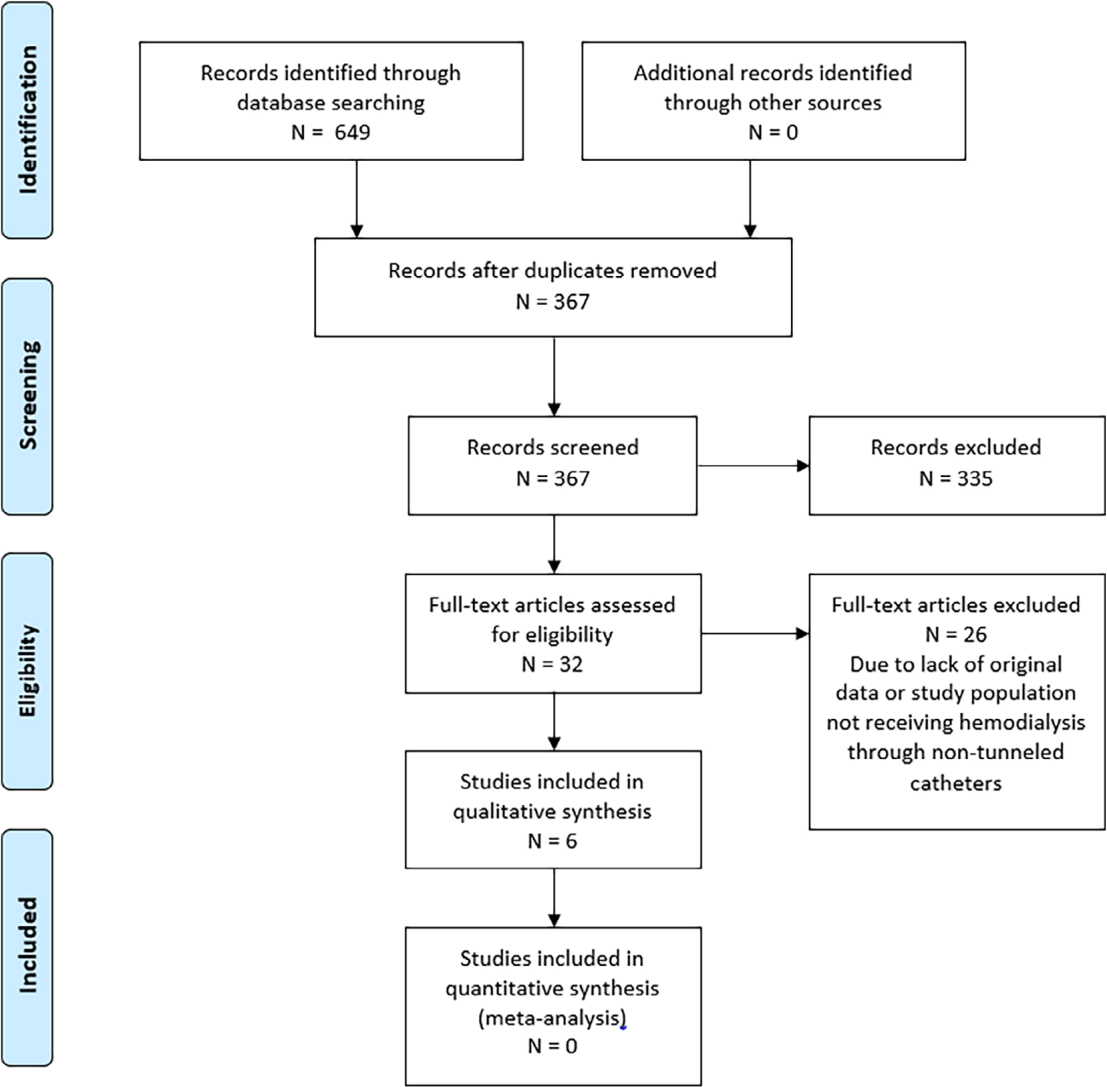
Flow chart of the selection of studies for inclusion in the review [Color figure can be viewed at wileyonlinelibrary.com]

We identified a total of 649 studies (Figure 1), namely 138 in PubMed, 473 in Cochrane and 38 in LiSSa. After identifying and removing duplicates, and screening titles and abstracts, 32 studies (4.9%) proceeded to full‐text screening. Twenty‐six studies were excluded for the following reasons: no original data (including systematic reviews), or the study population was not composed of patients receiving hemodialysis through non‐tunneled catheters. Finally, a total of six studies were included in the present review.^13^, ^14^, ^15^, ^16^, ^17^, ^18^

### Study characteristics

The characteristics of the studies included in the review are described in Table 1. Four of the six studies included were randomized trials,^13^, ^16^, ^17^, ^18^ one was a trial without randomization^14^ and one was a retrospective cohort study.^15^ Studies included between 78 and 1496 participants and were conducted in France,^13^, ^15^, ^16^, ^17^ China,^14^ Netherlands^18^ and Belgium.^18^ Half of the studies (n = 3) were multi‐center.^15^, ^17^, ^18^

**TABLE 1:** Characteristics of the studies included in the review of locking solutions for non‐tunneled hemodialysis catheters

Ref	Study characteristics	Participants	Intervention	Control	Catheter type	Outcomes	Results	
^13^	May 2009–August 2010 N = 78 Single‐center Randomization method not specified	Patients >18 years, requiring continuous or intermittent hemodialysis for acute renal failure hospitalized in the ICU	46.7% citrate solution	10 ml saline	Double‐lumen Polyurethane 13 Fr biocompatible 16 cm jugular 25 cm femoral	Catheter life span, catheter malfunction, catheter‐related bloodstream infection, predictors of catheter malfunction.	Citrate Catheter duration: 12 days Catheter dysfunction: 26 events/1000 catheter‐days (*p* < 0.00001)^a^ CRBSI: 24 infections/1000 catheter‐days	Saline Catheter duration: 6 days Catheter dysfunction: 127 events/1000 catheter‐days (*p* < 0.00001)^a^ CRBSI: 30 infections/1000 catheter‐days
^14^	January 2009–December 2016 N = 120 Single‐center No randomization, undefined method	Patients with AKI who had a central venous catheter, normal coagulation function and no bleeding tendency before catheterization, and chronic renal failure (combined with anuria or serum creatinine levels 707 mmol/L, serum potassium levels >6.5 mmol/L, or metabolic acidosis)	4% sodium citrate	6250, 5000 and 2500 IU/ml sodium heparin	Double‐lumen 12 Fr 16 cm femoral	Indicators of coagulation function were determined after treatment. Conduit blockage was identified. Hemorrhage Infection Leakage	Citrate Hematoma: 20% (*p* < 0.001)^a^ Thrombosis: 20% (*p* < 0.001)^a^ Infection: 13.2% (*p* < 0.001)^a^ Leakage: 26.8% (*p* < 0.001)^a^	Heparin 6250, 5000 and 2500 IU/ml Hematoma: 46.8%, 66.8% and 80.0% respectively (*p* < 0.001)^a^ Thrombosis: 26.8%, 60% and 70.0% respectively (*p* < 0.001)^a^ Infection: 36.8%, 50% and 63.2% respectively (*p* < 0.001)^a^ Leakage: 46.8%, 66.8% and 76.8% respectively (*p* < 0.001)^a^
^15^	January 2011–December 2012 N = 596 Multicenter (N = 11) Restrospective cohort study Randomization stratified by center for Cathedia study	Patients ≥18 years who were expected to require support with RRT. Only patients who were undergoing their first venous catheterization for acute RRT and without contraindications to attempt both jugular and femoral access were considered	Citrate 46.7%	Heparin and saline (standard of care)	Double‐lumen 14 Fr	Complications Catheter‐related infections Catheter‐related thrombosis	Citrate Catheter duration: 7.0 days CRBSI: 0.8% Catheter dysfunction: 3.0%	Heparin and saline: Catheter duration: 7.1 days CRBSI: 1.3% Catheter dysfunction: 9.9%
^16^	June 2013–January 2016 N = 402 (396 completed the trial) Single‐center Block randomization stratified by center	Patients >18 years, admitted to the ICU, with AKI requiring RRT, and in whom a first non‐tunneled hemodialysis catheter was to be inserted by the jugular or femoral vein, provided informed consent, and had social security coverage	4% trisodium citrate	UFH 5000 IU/ml	Double‐lumen 13.5 Fr 15 cm right jugular 20 cm left jugular 24 cm femoral	Primary endpoint: Duration of event‐free survival of the first non‐tunneled hemodialysis catheter. Secondary endpoints: fibrinolysis, catheter dysfunction, catheter‐related infection, catheter‐related bloodstream infections, hemorrhagic events requiring transfusion of at least two units of packed red blood cells, length of stay in intensive or critical care, length of hospital stay, death rate at 28 days.	Citrate Catheter duration: 7 days Bleeding at insertion site: 1% Hematoma: 2% Thrombosis: 5% Local CRI: 14% General CRI: 8% CRBSI: 2% Bleeding during follow‐up: 21% Catheter dysfunction: 15%	Heparin Catheter duration: 5 days Bleeding at insertion site: 3% Hematoma: 1% Thrombosis: 2% Local CRI: 19% General CRI: 2% CRBSI: 0 Bleeding during follow‐up: 19% Catheter dysfunction: 9.5%
^17^	June 2009–December 2011 N = 1496 Multicenter (N = 16) Randomization using a web‐based random‐number generator producing permuted blocks with stratification on ICU	Patients >18 years, who required insertion of a non‐tunneled, non‐antimicrobial‐impregnated double‐lumen DC with an expected duration of use longer than 48 h.	75% ethanol	0.9% saline	Double‐lumen	Primary endpoint: number of major CRI episodes per catheter. Secondary endpoints: frequencies of catheter colonization, severe mechanical complications, and adverse events.	Saline General CRI: 1.6% Catheter duration: 6.5 days Local CRI: 3.1% CRBSI: 1.3% Catheter dysfunction: 16%	Ethanol General CRI: 2.3% Catheter duration: 6.5 days Local CRI: 4.5% CRBSI: 1.2% Catheter dysfunction: 14.6%
^18^	April 2001–September 2002 N = 291 (193 non‐tunneled) Multicenter (N = 9) Computer generated randomization	Patients >18 years, were not admitted to the intensive care ward, and experienced chronic or acute renal failure that required hemodialysis treatment by means of a hemodialysis catheter	30% trisodium citrate	5000 IU/ml heparin	Left at the discretion of the interventional physician	CRB, catheter removal (any reason), exit‐site infection, removal for flow problem, adverse events, bleeding	Citrate Insertion site bleeding: 0.1% Bleeding during follow‐up: 0.6/1000 catheter‐days Local CRI: 6% General CRI: 0.7/1000 catheter‐days CRBSI: 5% Removal for any complication: 33% Catheter dysfunction: 24%	Heparin Insertion site bleeding: 0.3% Bleeding during follow‐up: 2.0/1000 catheter‐days Local CRI: 20% General CRI: 2.7/1000 catheter‐days CRBSI: 15% Removal for any complication: 48% Catheter dysfunction: 27%

^a^ Statistically significant differences.

A citrate‐containing locking solution was compared to heparin in four studies,^14^, ^15^, ^16^, ^18^ and to saline in one.^13^ In the Cathedia‐CLock study,^15^ the heparin control solution was combined with saline. In one study, ethanol was compared to a saline solution.^17^

### Results of individual studies

Event rates for individual studies are clustered in Table 1 for both primary and secondary outcomes.

### Primary outcomes

In all of the studies found, the duration of event‐free survival of the non‐tunneled catheter was between 5 and 7 days, except for the Citrate group in the first study^13^ that extended the survival to 12 days. The number of catheter related infections (CRI) tended to decrease when a citrate lock was used compared to other locking solutions but the results were only significant in one study.^14^ Adverse events such as bleeding or catheter dysfunction were significantly reduced in two studies when a citrate solution is used for catheter locking compare to saline^13^ or heparin.^14^

In the VERROUREA study,^16^ the primary endpoint was the duration of event‐free survival of the first non‐tunneled catheter, defined as the time from catheter insertion to withdrawal, whatever the reason. There was no significant difference between trisodium citrate 4% and heparin as a locking solution, with an overall event‐free duration of 7 days in the citrate group and 5 days in the heparin group. Souweine et al.^17^ and Parienti et al.^15^ also found that the locking solution did not impact on the duration of event‐free survival of the catheter. Souweine et al.^17^ chose the number of major CRI episodes per catheter as the primary outcome, with major CRI defined as either catheter‐related clinical sepsis without bloodstream infection (CRCS) or catheter‐related bloodstream infection (CRBSI). No significant difference was found between saline and ethanol locks. Three other studies^14^, ^16^, ^18^ reported that rates of CRI were lower with citrate than with heparin, but without reaching statistical significance. The risk of CRI was not significantly decreased either when citrate was used instead of saline^13^ or with a combination of saline and heparin.^15^

Arguments for removal of non‐tunneled catheters were described by Qureshi in a 2018 study.^19^ They found that 42.9% of catheters were removed prematurely due to malfunction, and 17% due to CRBSI.

### Secondary outcomes

Bleeding was defined as oozing or hematoma at the central venous catheter.^14^ There were no statistically significant differences between groups in the risk of bleeding for half of the studies.^14^, ^16^, ^18^ Only Huang et al.^14^ found significant results in favor of a citrate lock solution, with significantly lower rates of hematoma, conduit blockage, infection and leakage in the group with citrate 4% lock. All of the studies included reported either no significant difference between the different strategies, or else fewer catheter dysfunctions with a citrate lock.

## DISCUSSION

This is the first review of the literature to focus on catheter lock solutions for non‐tunneled hemodialysis catheter for the management of patients with acute kidney injury. It should be noted that it was not possible to perform meta‐analysis in view of the sparsity of available data, particularly for each different type of lock solution.

Indeed, studies comparing lock solutions use a range of different catheter locks, as well as different endpoints and definitions, be it for catheter dysfunction or infection. For example, in the VERROU‐REA study,^16^ catheter dysfunction was defined as the “inability to achieve and maintain a blood flow of more than 200 mL/min despite changing the patient's position, inverting the lines and flushing with saline solution”. Conversely, in the Cathedia study,^15^ it was defined as an inability to attain an adequate blood flow, requiring catheter replacement.

Secondary endpoints also differed between the studies included in the present review. In our opinion, the most pertinent of these endpoints to be taken into account is the bleeding rate. Indeed, heparin can leak into the circulation, causing systemic anticoagulation, which may incur a risk for intensive care patients. Bong et al.^20^ showed that the activated partial thromboplastin time (APTT) was considerably raised after heparin locking of catheter lumens. In the VERROUHEMOST study,^21^ Bovet et al reported that there was a median increase in anti‐Xa activity of 0.37 IU/ml (Q1; Q3: 0.20;0.67) after injection of the heparin lock solution (*p* < 0.0001), with an increase in anticoagulation levels in two‐thirds of samples, supporting the hypothesis that there is passage of heparin into the bloodstream after injection of a heparin lock.

Catheter dysfunction was also considered as in endpoint in many studies, and was numerically less frequent with citrate than saline or heparin in two studies.^13^, ^14^ However, the definitions were not exactly equivalent, rendering comparison between studies difficult. One of the most frequent complications is catheter‐related infection, and the US Center for Disease Control and Prevention (CDC) has published guidelines for their prevention.^22^ The use of prophylactic antimicrobial lock solutions is only recommended in patients with long term catheters who have a history of multiple CRBSI despite optimal maximal adherence to aseptic techniques. The practice of routine anticoagulant therapy is not recommended in the general ICU population. Another complication that may necessitate catheter removal is catheter obstruction, including thrombosis.

### Implications for clinical practice

Although the choice of catheter lock is important, factors related to the patient and comorbidities may also influence the risks and complications associated with the use of non‐tunneled catheters in the ICU. In a 2012 meta‐analysis,^23^ Parienti et al. found a link between increased body mass index (BMI) and catheter colonization. They also found that the use of a subclavian site versus alternative sites for central venous catheter insertion was associated with a significant reduction in CRI. In a more recent paper, Huriaux et al.^24^ recommend avoiding the subclavian site, as it may induce venous stenosis. The vascular access location is directly related to the catheter length, and therefore, a compromise must be found to maintain the best blood flow. The internal diameter also needs to be taken into account, as it is the main determinant of blood flow. In the studies in this review, catheters of different lengths and diameters were used, which may explain the discrepancies in results. However, double‐lumen catheters were used in all the studies included in the review. Double‐lumen catheters may increase the risk of thrombosis due to the large blood contact surface in the input line. This may be a limitation of these studies, even though double‐lumen catheters are widely used in the ICU.

In order to ensure that the catheter remains functional during the ICU stay of the patient, nurses have to carry out a number of tests, and follow a surgical asepsis process. Therefore, in addition to the mechanical risks of catheter failure detailed above, there also exists an infectious risk due to the repeated handling of the catheter. In this regard, it has been shown that maximal sterile barriers, such as the use of masks, caps, sterile gloves, gowns and drapes, have been shown to be effective in reducing catheter‐related infections.^22^, ^25^, ^26^ It is therefore of paramount importance that recommendations for asepsis be followed scrupulously in this context.

In terms of costs, there is a paucity of data comparing the costs (direct and/or indirect) arising from the use of citrate as compared to the use of heparin, which is considered as the reference. A medico‐economic evaluation is warranted, including micro‐costing, to estimate the costs of heparin preparation (syringes, needles, etc.) and compare it to ready‐to‐use citrate solutions. Citrate presents many advantages over heparin, including local anticoagulant and antiplatelet activity, absence of systemic anticoagulation, absence of HIT‐inducing potential, and antimicrobial activity, even as 4% trisodium citrate. In view of these advantages, citrate is recommended since 2019 by the Kidney Disease Outcomes Quality Initiative (KDOQI).^27^ It is particularly suited to prolonged use, as may be the case for hemodialysis via tunneled catheters, which may remain in place for months, even years. In theory, there is no excess risk in patients with acute kidney injury, particularly of metabolic disorders such as hypocalcemia,^21^ on condition that best practices are observed, notably slow injection using the quantities indicated on the catheter arms.

Compared to heparin, ethanol also presents some potential advantages as a lock solution, namely it is not expensive, it is widely available, and it is effective against a broad spectrum of bacteria and fungi.^28^, ^29^ Conversely, the duration of catheter ethanol exposure should be minimized, in order avoid ethanol‐induced catheter damage.^30^

This study has some limitations. Firstly, we identified only six studies for inclusion in our review, and data were insufficient to enable meta‐analysis. Secondly, outcomes and definitions differed across studies, rendering comparisons difficult.

In this review of the literature, we found only six studies investigating citrate versus other types of catheter lock solutions in patients with non‐tunneled hemodialysis catheters. There is a marked paucity of information in the literature, and existing data are too heterogeneous to enable recommending one type of catheter lock over any other. Large‐scale, prospective, randomized studies are needed to provide much‐needed data in this indication, and should include economic evaluations.

## AUTHOR CONTRIBUTIONS

Study conception and design: Isabelle Boucley, Jean‐Pierre Quenot; Data extraction: Isabelle Boucley, Jean‐Pierre Quenot, Marine Jacquier, Amélie Cransac. Data analysis and interpretation: Isabelle Boucley, Auguste Dargent, Pascal Andreu, Jean‐Baptiste Roudaut, François Aptel, Marie Labruyère, Marine Jacquier, Amélie Cransac, Jean‐Pierre Quenot; Drafting the manuscript: Isabelle Boucley, Jean‐Pierre Quenot; all authors read and approved the final manuscript.

## CONFLICT OF INTERESTS

No author has any conflict of interest to disclose.
